# An Alcohol Symptom Checklist identifies high rates of alcohol use disorder in primary care patients who screen positive for depression and high-risk drinking

**DOI:** 10.1186/s12913-022-08408-1

**Published:** 2022-09-05

**Authors:** Emma D. Ryan, Yanni M. Chang, Malia Oliver, Katharine A. Bradley, Kevin A. Hallgren

**Affiliations:** 1 University of Washington School of Medicine, 1959 NE Pacific St, Seattle, WA 98195 USA; 2 Department of Psychiatry and Behavioral Sciences, University of Washington, Seattle, WA USA; 3 Kaiser Permanente Washington Health Research Institute, Seattle, WA USA; 4 Department of Health Systems and Population Health, University of Washington, Seattle, WA USA; 5 Department of Medicine, University of Washington, Seattle, WA USA

**Keywords:** Alcohol use disorder, Alcohol screening, Brief intervention, Depression, Population health, Primary care

## Abstract

**Background:**

Although alcohol use disorder can complicate depression management, there is no standard process for assessing AUD *symptoms* (i.e., AUD diagnostic criteria) in primary care for patients who screen positive for depression. This study characterizes the association between depressive symptoms and high-risk drinking reported by primary care patients on screening measures in routine care. Then, using data from a novel clinical program, this study characterizes the association between depressive symptoms and AUD symptoms reported by primary care patients with high-risk drinking via an Alcohol Symptom Checklist.

**Methods:**

In this cross-sectional study, electronic health record data were obtained from patients who visited 33 Kaiser Permanente Washington primary care clinics between 03/2018 and 02/2020 and completed depression (PHQ-2) and alcohol consumption (AUDIT-C) screening measures as part of routine care (*N* = 369,943). Patients who reported high-risk drinking (AUDIT-C scores 7–12) also completed an Alcohol Symptom Checklist where they reported the presence or absence of 11 AUD criteria as defined by the DSM-5 (*N* = 8,184). Generalized linear models estimated and compared the prevalence of high-risk drinking (AUDIT-C scores 7–12) and probable AUD (2–11 AUD symptoms on Alcohol Symptom Checklists) for patients with and without positive depression screens.

**Results:**

Patients who screened positive for depression had a 131% higher prevalence of high-risk drinking than those who screened negative (5.2% vs. 2.2%; *p* < 0.001). Among patients with high-risk drinking, positive depression screens were associated with a significantly higher prevalence of probable AUD (69.8% vs. 48.0%; *p* < 0.001), with large differences in the prevalence of probable AUD observed with increasing PHQ-2 scores (e.g., probable AUD prevalence of 37.6%, 55.3% and 65.2%, for PHQ-2 scores of 0, 1, and 2, respectively). Although the overall prevalence of high-risk drinking was higher for male patients, similar patterns of association between depression screens, high-risk drinking, and AUD symptoms were observed for male and female patients.

**Conclusions:**

Patients with positive depression screens are more likely to have high-risk drinking. Large percentages of patients with positive depression screens and high-risk drinking report symptoms consistent with AUD to healthcare providers when given the opportunity to do so using an Alcohol Symptom Checklist.

**Supplementary Information:**

The online version contains supplementary material available at 10.1186/s12913-022-08408-1.

## Background

Alcohol use disorder (AUD) is the most prevalent substance use disorder in the United States and commonly co-occurs with depression [1]. Depression is also common (occurring in an estimated 8.4% of US adults) [2], and an estimated 16–25% of people with major depression have AUD [1, 3]. Co-occurring AUD and depression complicate each other and are associated with higher morbidity, mortality, functional impairment, and suicide risk compared to when either of these conditions occur alone [4–6]. Although patients with co-occurring depression and AUD can benefit from depression treatment alone [7], treating both AUD and depression concurrently with medications or behavioral treatments may increase the likelihood of reduced drinking and depressive symptoms [8, 9].

Screenings for depression and unhealthy alcohol use using validated instruments like the Patient Health Questionnaire (PHQ)-2 and Alcohol Use Disorders Identification Test-Consumption version (AUDIT-C) have been increasingly implemented in adult primary care settings [10–12]. However, very few health systems systematically assess whether patients meet criteria for an AUD diagnosis as defined by the Diagnostic and Statistical Manual for Mental Disorders (DSM-5), even when high-risk drinking or depression is detected on screening measures. A diagnosis of AUD requires patients to meet at least 2 out of 11 DSM-5 AUD criteria within the past year (e.g., inability to cut down or control drinking, drinking larger amounts or for longer than intended, tolerance, withdrawal, continued drinking despite the negative impact of drinking on health or relationships, etc.), and an AUD diagnosis cannot be determined from screening measures that ask solely about alcohol consumption (e.g., the AUDIT-C) or from screening measures that only inquire about a few AUD criteria (e.g., the full 10-item AUDIT, the Alcohol, Smoking and Substance Involvement Screening Test [ASSIST]) [13].

Referral to specialty AUD treatment from primary care has not been shown to increase receipt of AUD treatment [14]. However, eliciting symptoms of AUD and engaging patients regarding treatment options in primary care may increase AUD diagnosis, precipitate changes drinking [15], and guide treatments [16, 17] including medications for AUD that can be prescribed from primary care to patients with AUD and/or behavioral interventions that may specifically target symptoms of AUD. To help facilitate symptom assessment, patient engagement, diagnosis, and treatment, Kaiser Permanente Washington (KPWA), an integrated health system in Washington State, began implementing routine annual integrated mental health care that included screening for depression symptoms (using the PHQ-2) and unhealthy alcohol use (using the AUDIT-C) for all adult primary care patients. For patients who screened positive for “high-risk drinking” (defined by an AUDIT-C score of 7–12, regardless of the PHQ-2 depression screening score), these screens were followed by standardized assessments of DSM-5 AUD criteria using patient-report Alcohol Symptom Checklist questionnaire [18]. These screening and assessment measures were implemented as part of a system-wide effort to integrate behavioral healthcare across all primary care sites, with the goal of helping providers detect depression, unhealthy alcohol use, and AUD in patients with high-risk drinking in order to facilitate treatment [18–21]. Alcohol Symptom Checklists have recently been recommended for eliciting patient-reported information about AUD symptoms, facilitating clinical discussion about potential negative effects of drinking, and guiding shared decision-making about alcohol-related treatment options [16, 17, 22].

### Aims

Integrating alcohol-related screening and assessment with other mental health screening in primary care could help increase identification of co-occurring depression, high-risk drinking, and AUD. However, little is known about how often primary care patients with depression would report high-risk drinking or symptoms of AUD to healthcare providers when given the opportunity to do so on screening and assessment instruments that are integrated into routine care. Using screening and assessment data completed by primary care patients under real-world routine-care conditions, the current study aims to evaluate the association between depression screening scores and (1) the prevalence of high risk drinking and (2) the prevalence of probable AUD among patients with high-risk drinking. We hypothesized, based on epidemiologic research conducted outside of real-world routine care settings [1, 23], that depression symptoms reported on real-world routine care screening measures would be associated with an increased prevalence of high-risk drinking and DSM-5 AUD symptoms reported on screening and assessment measures completed as part of routine care.

## Methods

### Study setting

This cross-sectional study analyzed electronic health record (EHR) data of patients who received primary care at Kaiser Permanente Washington (KPWA). KPWA is a not-for-profit health system that offers health insurance and integrated primary care and specialty care across Washington State in the United States. Data were obtained from patients seen at KPWA’s 33 primary care clinics who completed validated depression and alcohol screening measures between March 1, 2018 and February 29, 2020. This study’s use of existing clinical data for research purposes was approved by the KPWA Health Research Institute’s Institutional Review Board with a waiver of consent and Health Information Portability and Accountability Act authorization.

### Screening and assessment procedures

Beginning in 2015, KPWA implemented annual behavioral health screening for all adult primary care patients that included brief screens for depression and unhealthy alcohol use [18, 23]. In primary care settings where most screens were completed, the KPWA EHR automatically prompted check-in staff and medical assistants to administer paper screening questionnaires if they had not been completed within the past year. Medical assistants then entered the results into the EHR, typically before the patient was seen by the primary care provider. During the study observation period, these screens were completed by 88% of all adult patients who had primary care visits. If the alcohol screen indicated high-risk drinking (defined below), the EHR prompted the medical assistant to give the patient a paper Alcohol Symptom Checklist that assessed whether the patient experienced each of the 11 criteria for AUD (described below). Depression screening scores did *not* factor into whether the EHR prompted medical assistants to administer Alcohol Symptom Checklists. After patients completed the Alcohol Symptom Checklist, the results were entered into the EHR prior to the visit with the provider. Providers could then utilize the information on the Alcohol Symptom Checklist to guide clinical discussions about AUD symptoms, determine if an AUD diagnosis is present, and facilitate shared decision making around AUD treatment options.

### Patient population

Our analyses utilized two samples of patients: a screening sample consisting of patients who completed screens for both depression and unhealthy alcohol use (via the PHQ-2 and AUDIT-C, respectively), and a nested subsample comprised of patients from the screening sample who reported high-risk drinking on the AUDIT-C (AUDIT-C scores 7–12) and subsequently completed an Alcohol Symptom Checklist on which they reported the presence or absence of AUD symptoms. Eligibility criteria for the screening sample included (a) having at least one visit to a KPWA primary care clinic during the study period, (b) completing depression and alcohol screens during the study period, and (c) being 18 years or older at the time the screening. Eligibility criteria for the checklist sample further required (d) reporting high-risk drinking on the alcohol screen (AUDIT-C score 7–12) and (e) completing an Alcohol Symptom Checklist.

### Measures

#### Depression screening measure

The PHQ-2 is a two-item self-report depression screening questionnaire that is included in the annual behavioral health screen completed by most KPWA primary care patients [24, 25]. The PHQ-2 consists of the first two items of the longer PHQ-9 measure [26], and asks about the frequency of depressed mood and anhedonia over the past two weeks. Response options range from “not at all” (0) to “nearly every day” (3) and responses to the two items are summed to create a total score ranging from 0–6. Psychometric analyses indicate that the PHQ-2 has slightly lower sensitivity but similar specificity as the longer, 9-item PHQ-9 [24, 27]. The PHQ-9 was not included in the current study; however, the briefness of the two-item PHQ-2 measure often makes it a more practical measure for universal screening of depression in many medical settings [28, 29]. PHQ-2 scores ≥ 3 have been validated as a screen for major depressive disorder with moderate sensitivity (0.61) and high specificity (0.92) [20] and are commonly used in healthcare settings to indicate a positive depression screen. Therefore, in the current study, we considered depression screens to be positive when PHQ-2 scores were ≥ 3. However, PHQ-2 scores ≥ 2 have higher sensitivity (0.86) and lower specificity (0.78) [24] and may be used as an alternative screening threshold for positive PHQ-2 screens, so we performed supplemental analyses using this lower cutoff to indicate positive depression screens (see eSupplement).

#### Alcohol screening measure

The AUDIT-C is a validated 3-item self-report alcohol screening questionnaire that is included in the annual behavioral health screen completed by most KPWA primary care patients [27–29]. The AUDIT-C includes questions about the frequency of alcohol consumption, the typical number of drinks per drinking day, and the frequency of heavy drinking episodes (≥ 6 drinks within a single occasion). Of note, the AUDIT-C does not include any questions about DSM-5 criteria for AUD, and an AUD diagnosis requires the presence of DSM-5 AUD symptoms but does not require any particular level of alcohol consumption that can be measured by the AUDIT-C [30]. Response options for each item on the AUDIT-C are scored from 0–4, yielding a total score from 0–12 with average alcohol consumption and AUD symptoms increasing as scores increase [31]. While ≥ 3 or ≥ 4 points is typically used as the threshold for offering *preventive*counseling in women and men respectively (i.e. brief interventions to reduce potentially harmful levels of alcohol consumption) [32], patients with scores ≥ 7 were considered to have high-risk drinking in the KPWA health system due to these scores being associated with substantially higher average daily alcohol consumption and risk for AUD in the general US population [31]. Although the AUDIT-C does not assess AUD criteria, scores on the AUDIT-C correlate strongly with risk for AUD [31–34]. The AUDIT-C has been shown to have good sensitivity and specificity as a screener for AUD [31–33], including in primary care patients [32] and in populations with mood disorders [35], and the 3-item AUDIT-C has similar sensitivity and specificity for detecting AUD as the longer 10-item AUDIT questionnaire [32, 33]. Therefore, the AUDIT-C is recommended as a first-line screener for identifying individuals who drink at unhealthy levels, many of whom may have AUD [36]. However, additional assessment is required for patients who screen positive for high-risk drinking to assess whether they meet specific criteria for AUD.

#### Alcohol Symptom Checklist

In KPWA, primary care patients with high-risk drinking are asked to complete an Alcohol Symptom Checklist as part of routine care to assess whether symptoms of AUD are also present [31, 37]. The Alcohol Symptom Checklist [38, 39] is an 11-item self-report questionnaire that asks patients whether they have experienced each of the 11 AUD criteria within the past year. Each of the 11 items on the Alcohol Symptom Checklist maps onto one the 11 criteria for AUD as currently defined by the DSM-5 [40]. Patients indicate whether each AUD criterion was present or absent within the past year and Alcohol Symptom Checklist scores reflect AUD criteria counts that range from 0–11. Endorsing 2–3 criteria, 4–5 criteria, or 6–11 criteria is consistent with DSM-5 definitions for mild, moderate, or severe AUD, respectively. Psychometric analyses have supported the reliability [39] and validity [38] of the Alcohol Symptom Checklist, showing that it measures AUD criteria consistently over time [39], along a unidimensional continuum of severity (consistent with DSM-5 conceptualization) [38, 39], and similarly across sex, age, race, and ethnicity subgroups when it is completed in routine care by patients with high-risk drinking [38].

#### Demographics

Demographic measures, including age, sex, race, and ethnicity, were obtained from EHR data and used as covariates in regression models (described in Analytic Approach). Race and ethnicity were coded to align with categories defined by the United States National Institutes of Health.

#### Analytic approach

Data were analyzed cross-sectionally to evaluate the prevalence of high-risk drinking (i.e., AUDIT-C scores between 7–12), probable AUD (i.e., Alcohol Symptom Checklist scores between 2–11), and the associations of depression screening scores with high-risk drinking and AUD symptoms. When patients had multiple screens or multiple Alcohol Symptom Checklists, only the first completed screen or checklist was retained to allow cross-sectional analyses with independent observations.

### Screening analyses

Descriptive statistics were used to characterize patient demographics and screening results among all patients in the screening sample. Generalized linear models (GLMs) were then used to evaluate the associations of depression screening scores (predictor) with prevalence of high-risk drinking (outcome), controlling for the demographic covariates described above. In the GLMs, the dependent variable was a binary indicator of high-risk drinking being present (i.e., AUDIT-C score 7–12) or absent (i.e., AUDIT-C score 0–6). Independent variables in the GLMs included depression screening scores (predictor) and demographic measures (covariates entered in analyses to statistically adjust for age, sex, race, and ethnicity).

We performed two separate sets of analyses, including analyses where (1) the depression screen (predictor) was treated as a scaled, continuous measure (i.e., PHQ-2 score ranging from 0 to 6) and where (2) the depression screen (predictor) was treated as a binary measure (i.e., PHQ-2 scores ≥ 3 coded as positive). These two sets of analyses were conducted to reflect two ways that the PHQ-2 may be used clinically, including (1) as a scaled measure of depression symptom severity and/or (2) as a screening tool where a binary cutoff is used to indicate whether depression is likely present or absent. The AUDIT-C (dependent variable in GLMs) was always treated as a binary variable indicating whether high-risk drinking was present (1) or absent (0). GLMs used a Poisson link function with robust “sandwich” error estimation to obtain adjusted prevalence ratios (aPRs) of high-risk drinking across depression screening scores. When modified Poisson regression with robust “sandwich” error estimation is used with a binary outcome, the resulting regression coefficients can be directly transformed into adjusted prevalence ratios [41]. In contrast, when logistic regression is used [which we did not use in the current study], the resulting regression coefficients are typically transformed into adjusted odds ratios. We utilized modified Poisson regression in this study (rather than logistic regression) because prevalence ratios are more often correctly interpreted by researchers and clinicians, whereas odds ratios are commonly misinterpreted [41]. For analyses that used the PHQ-2 as a scaled score (0–6), the estimated aPRs reflect the relative increase in the prevalence of high-risk drinking for each one-point increase in depression screening scores, adjusting for demographics. For analyses that used the PHQ-2 as a binary score (0–2 vs. 3–6), the estimated aPRs reflect the relative increase in the prevalence of high-risk drinking for patients with positive depression screens compared to patients with negative depression screens, controlling for demographics. Supplemental analyses that stratified by patient sex also were performed.

### Alcohol Symptom Checklist analyses

Descriptive statistics characterized patient demographics, screening results, and probable AUD (mild, moderate, or severe) in patients with high-risk drinking who completed Alcohol Symptom Checklists (i.e., checklist sample). Similar to the screening sample analyses, GLMs were used to estimate the prevalence of probable AUD (binary outcome equal to 1 if Alcohol Symptom Checklist scores 2–11 vs. 0 if Alcohol Symptom Checklist scores 0–1) based on depression screening scores (using PHQ-2 scores as both a scaled and a binary measure), adjusting for demographics among patients in the checklist sample. Models used a two-tailed alpha-level of 0.017 (i.e., 0.05/3 to adjust for PHQ-2 scores being modeled in three ways: binary cutoff ≥ 3, binary cutoff ≥ 2, and as a scaled score 0–6). Analyses were performed in R [42] with the *sandwich* [43] and *emmeans* [44] packages for computing sandwich errors and marginal mean estimates, respectively.

## Results

### Description of samples

There were 369,943 patients who met study criteria and were included in the screening sample analyses. The screening sample (see Table 1) was predominantly female (58.6%), white (72.1%), and non-Hispanic (88.8%).

**Table 1:** Descriptive Statistics for Screening Sample and Alcohol Symptom Checklist Sample

		Screening Sample (*N* = 369,943)		Alcohol Symptom Checklist Sample (*N* = 8,184)	
		N	%	N	%
Sex	Male	152,994	41.4%	5,652	69.1%
	Female	216,947	58.6%	2,532	30.9%
Race	Asian or Asian American	35,625	9.6%	387	4.7%
	Black or African American	17,383	4.7%	401	4.9%
	Native Hawaiian/Pacific Islander	3,521	1.0%	90	1.1%
	American Indian/Alaska Native	2,581	0.7%	81	1.0%
	White	266,715	72.1%	6,171	75.4%
	More than one race	10,363	2.8%	221	2.7%
	Other race	14,788	4.0%	395	4.8%
	Unknown	18,967	5.1%	438	5.4%
Ethnicity	Hispanic	22,164	6.0%	578	7.1%
	Non-Hispanic	328,405	88.8%	7,146	87.3%
	Unknown	19,374	5.2%	460	5.6%
Age	18–24	31,232	8.4%	833	10.2%
	25–44	117,296	31.7%	3551	43.4%
	45–64	132,896	35.9%	2905	35.5%
	65 +	88,519	23.9%	895	10.9%
AUDIT-C score	0 (no-past year drinking)	105,486	28.5%	–-	–-
	1–2 (F) or 1–3 (M) (low-level drinking)	159,057	43.0%	–-	–-
	3–4 (F) or 4 (M) (mild UAU^a^)	69,886	18.9%	–-	–-
	5–6 (moderate UAU^a^, no checklist)	25,852	7.0%	–-	–-
	7–12 (high-risk drinking, checklist)	9662	2.7%	8184	100%
Alcohol Symptom Checklist	0 (no AUD symptoms)			2,546	31.1%
	1 (no AUD)			1,215	14.8%
	2–3 (mild AUD)			1,631	19.9%
	4–5 (moderate AUD)			980	12.0%
	6–11 (severe AUD)			1,812	22.1%
PHQ-2 score	0–2 (negative depression screen)	321,869	87.3%	5,903	72.2%
	3–6 (positive depression screen)	47,025	12.7%	2,278	27.8%

*Note*: ^a^ *UAU* Unhealthy alcohol use

Based on AUDIT-C alcohol screening, 28.5% of the screening sample reported no past-year drinking (AUDIT-C = 0), 43.0% reported low-level drinking (AUDIT-C = 1–2 [women] or 1–3 [men]), 25.9% reported mild to moderate unhealthy alcohol use (AUDIT-C = 3–6 [women] or 4–6 [men]), and 2.7% reported high-risk drinking (AUDIT-C = 7–12). Adult primary care patients who were *not* included in the current analyses because they did not complete the PHQ-2 and AUDIT-C screening measures are characterized in eTable 1 (supplement; *n* = 50,818); although they were largely similar in demographics, adult primary care patients who were *not* included in the analysis were slightly more likely to be male, younger, and have an unknown race or ethnicity compared to patients who were included.

There were 8,184 patients who had high-risk drinking on the AUDIT-C and completed an Alcohol Symptom Checklist (i.e., 84.7% of patients with high-risk drinking). This checklist subsample was predominantly male (69.1%), white (75.4%), and non-Hispanic (87.3%). In the checklist subsample, 31.1% reported no AUD symptoms and 14.8% reported one AUD symptom (below threshold for AUD), whereas 19.9% reported 2–3 symptoms (consistent with mild AUD), 12.0% reported 4–5 symptoms (consisted with moderate AUD), and 22.1% reported 6–11 symptoms (consistent with severe AUD). Patients with high-risk drinking who did *not* complete the Alcohol Symptom Checklist are characterized in eTable 2 (supplement, *n* = 2,851); although they were largely similar in demographics, those who did *not* complete the Alcohol Symptom Checklist were slightly more likely to be male, Asian/Asian American, multiracial, have an unknown race, and have an unknown ethnicity compared to those who completed the Alcohol Symptom Checklist.

### Prevalence of high-risk drinking across depression screens

**Fig. 1:**
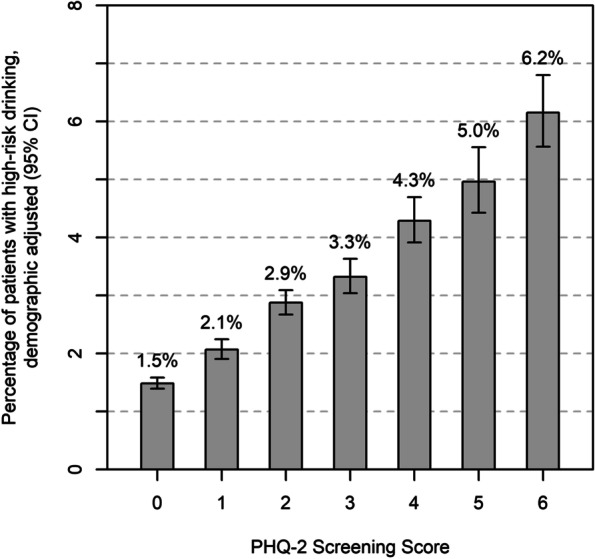
Prevalence of high-risk drinking (AUDIT-C score ≥ 7) across PHQ-2 depression screening scores in the screening sample, adjusted for demographics (*N* = 369,943)

High-risk drinking was strongly associated with depression screens completed in routine care, including when PHQ-2 depression screens were analyzed as a scaled or binary measure. When the PHQ-2 was used as a scaled measure, the prevalence of high-risk drinking increased across the range of depression screening scores from 1.5% (when PHQ-2 score = 0) to 6.2% (when PHQ-2 score = 6; see Fig. 1), adjusting for demographics. Although male patients had a higher prevalence of high-risk drinking compared to female patients, the association was similar in male and female patients: prevalence ranging from 3.1% to 9.2% in male patients and 0.6% to 4.4% in female patients (eFigure 1 in supplement).

Adjusting for demographics, the prevalence of high-risk drinking in the screening sample increased by 27% for every one-point increase on depression screening scores (aPR = 1.27, 95% CI: 1.26–1.29, *p* < 0.001; see supplemental eTable 3 for complete regression model results). When the PHQ-2 was used as a binary measure, the prevalence of high-risk drinking was 131% higher among patients who screened positive for depression (5.2%) compared to patients who screened negative for depression (2.2%; aPR = 2.31, 95% CI: 2.21–2.42, *p* < 0.001), adjusting for demographics (see supplemental eTable 4 for complete regression model results). Patterns of results were similar when the PHQ-9 had a cutoff score of 2 instead of 3 (see supplemental eTable 5).

### Prevalence of probable DSM-5 AUD across depression screens

**Fig. 2:**
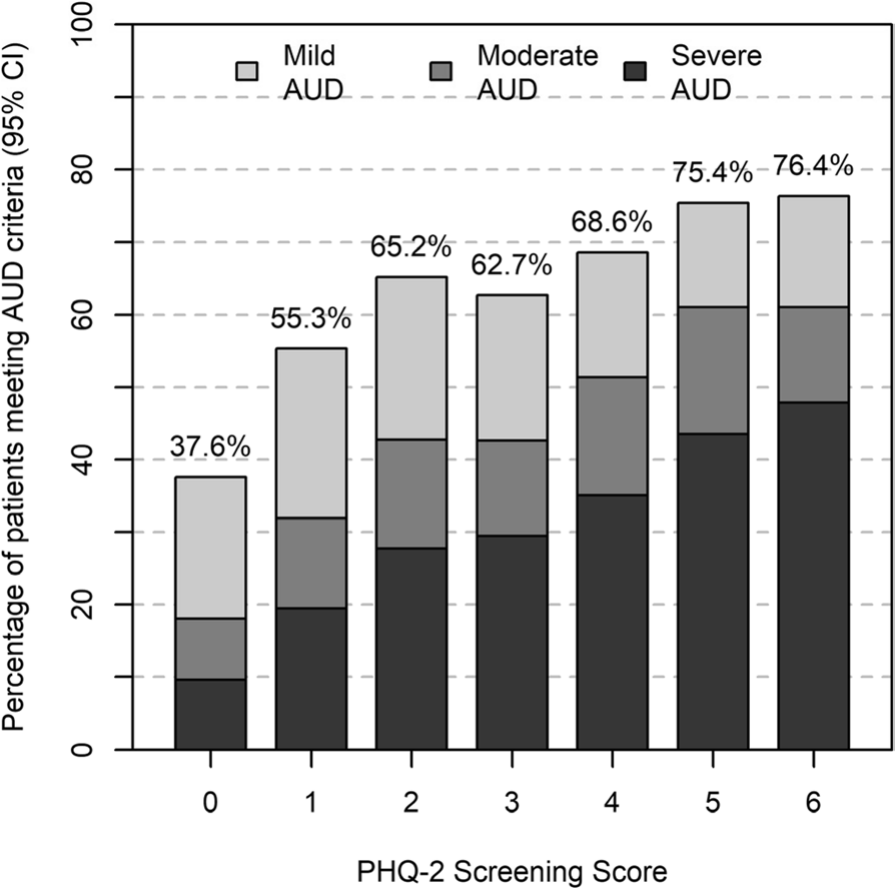
Prevalence of probable AUD (mild, moderate, or severe) based on Alcohol Symptom Checklists completed by patients in routine care (*N* = 8,184 patients with high-risk drinking in the checklist sample)

AUD symptoms were strongly associated with depression screening scores in patients with high-risk drinking, including when PHQ-2 depression screens were analyzed as a scaled or binary measure. When the PHQ-2 was used as a scaled measure, the prevalence of probable AUD (Alcohol Symptom Checklist scores 2–11) increased most sharply as depression screening scores increased 0 to 2, from 37.6% (when PHQ-2 score = 0) to 65.2% (when PHQ-2 score = 2). The prevalence of probable AUD increased less sharply as depression screening scores increased from 3 to 6, from 62.7% (which PHQ-2 score = 3) to 76.4% (when PHQ-2 score = 6; see Fig. 2).

Adjusting for demographics, the prevalence of probable AUD increased by 12% (aPR = 1.12, 95% CI: 1.11–1.13, *p* < 0.001) for every one-point increase in PHQ-2 depression screening scores (see supplemental eTable 6 for complete regression model results). This association was principally driven by an 11% increase in the adjusted prevalence of probable moderate AUD (aPR = 1.11, 95% CI: 1.07–1.14, *p* < 0.001) and a 26% increase in the adjusted prevalence of probable severe AUD (aPR = 1.26, 95% CI: 1.24–1.29, *p* < 0.001) for every one-point increase in PHQ-2 depression screening scores (see Fig. 2). When the PHQ-2 was used as a binary measure, the prevalence of probable AUD was 42% higher among patients who screened positive for depression (69.8%) compared to patients who screened negative for depression (48.0%; aPR = 1.42, 95% CI: 1.36–1.47, *p* < 0.001), adjusting for demographics (see supplemental eTable 7 for complete regression model results). Patterns of results were similar when the PHQ-2 had a cutoff score of 2 instead of 3 (see supplemental eTable 8 – note: these results were obtained from the symptom checklist subsample, where all patients had already screened positive for high-risk drinking.) Similar patterns of results were obtained for male and female substrata (see supplemental eFigure 2).

## Discussion

The current study characterized the prevalence of high-risk drinking in patients who reported depression symptoms and the prevalence of AUD symptoms in primary care patients who reported both high-risk drinking and depression – all based on practical measures that were implemented as part of routine care within an integrated health system. Results indicated that primary care patients who screened positive for depression in routine care had more than a two-fold increased prevalence of reporting high-risk drinking on the AUDIT-C compared to patients who did not screen positive for depression (5.2% vs. 2.2%, respectively). Moreover, among primary care patients who reported high-risk drinking, those who screened positive for depression had more than a 40% higher prevalence of probable AUD based on Alcohol Symptom Checklist scores compared to patients who did not screen positive for depression (69.8% vs. 48.0%, respectively). The increased prevalence of high-risk drinking and probable AUD among people with depression is consistent with previous epidemiological research conducted outside of routine care settings with longer measures that are typically impractical to use in routine care [3].

In routine care settings, identifying AUD symptoms when they co-occur with depression is important for effectively addressing both AUD and depression [4–6, 8]. This is the first study to our knowledge to characterize the prevalence of high-risk drinking and AUD symptoms reported as part of routine care to primary care patients with varying depression screening scores. This large primary care study demonstrates that commonly used screening questionnaires for depression and unhealthy alcohol use—the PHQ-2 and AUDIT-C—can be used to identify patients who could benefit from co-management of depression and AUD.

Primary care practices increasingly screen for unhealthy alcohol use (e.g., using lower AUDIT-C score cutoffs of ≥ 3 or ≥ 4 points) [40] to identify the 15–25% of patients who could potentially benefit from preventive brief interventions [18, 36, 45]. However, direct assessment of AUD symptoms is less common within primary care [46] and the AUDIT-C and other alcohol screening measures typically do not provide the information necessary to diagnose AUD as defined by the DSM-5, potentially hindering the detection, diagnosis, and treatment AUD within primary care despite the availability of medications and behavioral treatments for AUD that can be offered within primary care [47, 48]. The results of the current study indicate that many patients with high-risk drinking—especially those with depression symptoms—are willing to report AUD symptoms in routine care settings when given the opportunity to do so using an Alcohol Symptom Checklist. In contrast, settings that do not use Alcohol Symptom Checklists may miss opportunities to detect, diagnose, and treat AUD even when it is present, including for a high percentage of patients who also screen positive for depression—many of whom may be receiving depression treatment that is complicated by AUD symptoms.

The current findings highlight the value of implementing routine, universal alcohol screening as part of integrated behavioral health care for all adult primary care patients to detect high-risk drinking *and*following those screens by structured assessment of AUD symptoms using an Alcohol Symptom Checklist when patients screen positive for high-risk drinking. As reported here, KPWA’s implementation of routine screening for alcohol and depression resulted in most adult patients completing these screens (88%), and most patients who screened positive for high-risk drinking also completed an Alcohol Symptom Checklist (84.7%) [18].

Little is known about the association between the *severity* of depression symptoms as reported on scaled depression screening questionnaires and the prevalence of symptoms of AUD in primary care populations. This study found a graded association between PHQ-2 scores (0–6) and the prevalence of both high-risk drinking and AUD symptoms. In primary care patients, the prevalence of high-risk drinking increased almost linearly from 1.5% to 6.2% as depression scores increased from 0 to 6. However, among patients with high-risk drinking, the prevalence of probable AUD increased sharply as PHQ-2 scores increase from 0 to 2 (from a prevalence of 37.6% to 65.2%), then less sharply as PHQ-2 scores increase from 3 to 6 (from a prevalence of 62.7% to 76.4%), with similar patterns for male and female patients. This suggests that the increased prevalence of AUD may be present even with relatively mild depression symptoms (i.e., PHQ-2 scores of 1 or 2). However, future studies are needed in patients who complete the longer PHQ-9 depression screen [25] which provides more detailed information about depression symptoms (i.e., 9 depression symptoms assessed) and provides a wider continuum of depression severity (scores range from 0 to 27).

The results of the current study also may suggest that many patients who screen positive for depression could benefit from a lower threshold for assessing AUD symptoms, for example, even if their AUDIT-C scores are lower than 7. Because it was not feasible to administer Alcohol Symptom Checklists to all patients with potentially unhealthy levels of drinking (i.e., AUDIT-C scores ≥ 3 or ≥ 4), the KPWA health system selected a higher AUDIT-C score threshold of ≥ 7 for administering paper Alcohol Symptom Checklist questionnaires, as this is also a threshold at which drinking and AUD symptoms increase substantially within the general population [31, 34, 35]. However, for patients in the checklist sample (AUDIT-C scores 7–12), the prevalence of probable AUD was considerably higher when depression screens were positive (i.e., 69.8% prevalence of probable AUD among patients with positive depression screens and AUDIT-C scores 7–12), suggesting that a lower AUDIT-C score threshold might be warranted for triggering assessment with the Alcohol Symptom Checklist in primary care patients with depression symptoms. For example, it is possible that AUDIT-C score thresholds of ≥ 3 or ≥ 4, which are commonly used as thresholds for brief alcohol interventions, may be better suited for triggering AUD symptom assessments in patients who also screen positive for depression. However, we could not directly test this hypothesis in this study. Future studies may evaluate the impact of reducing the threshold for AUD symptom assessment in patients with positive depression screens.

### Limitations and strengths

This study had noteworthy limitations. First there are design limitations, including that the study was cross-sectional and relied on secondary data that was collected for clinical use rather than for research. Therefore, the results should not be interpreted as indicating whether depression, alcohol consumption, and AUD symptoms are causally related, although previous studies suggest there are likely dynamic and bidirectional relations between alcohol use and depression symptoms [49]. Second, there are sampling limitations, including that all patients in the study were insured members of KPWA and therefore results may not generalize to uninsured populations, many of whom may be socioeconomically disadvantaged which in turn may affect their depression, alcohol consumption, and access to care for these concerns differently than patients in the current sample. Patients in the checklist subsample were predominantly white and male, and although this reflects the racial/ethnic makeup of Washington state (predominantly white) and individuals with high-risk drinking (predominantly male), caution is still warranted in generalizing findings to diverse populations including people of color who comprise relatively small percentages of the current sample. Primary care patients who were male, younger, or had unknown race/ethnicity were slightly less likely to complete the AUDIT-C, and patients who were male, Asian/Asian American, multiracial, or had unknown race/ethnicity were slightly less likely to complete the Alcohol Symptom Checklist. Given that younger males tend to consume higher quantities of alcohol than other demographics, it may be that our study underestimates the prevalence of high-risk drinking and/or AUD. Finally, there are measurement limitations, including that Alcohol Symptom Checklists are typically only completed by KPWA patients who screen positive for high-risk drinking (i.e., AUDIT-C scores 7–12). Although previous studies show that patients with AUDIT-C scores in this range have the highest risk for AUD [31], it is possible that patients with lower AUDIT-C scores could report AUD symptoms that would not be detected within the KPWA data utilized here, including for patients with depression symptoms. Future research should evaluate the prevalence of AUD symptoms in patients with lower AUDIT-C scores, including for patients with and without depression symptoms. Additionally, although results for male and female patient subgroups were described in supplemental analyses (eFigures 1 and 2), differences between male and female subgroups were not statistically evaluated; therefore, future research may more comprehensively evaluate differences based on sex and other demographic factors (e.g., race, socioeconomic status).

The study also has noteworthy strengths. Our use of data collected for real-world routine care allowed us to evaluate the performance of practical screening and assessment measures as they actually function when used in real-world health care settings. This provides particularly strong external validity of the findings, as the results obtained here could be expected to represent the results of depression and alcohol measures completed by patients in real-world routine care contexts as opposed to measures completed in research contexts. Our inclusion of all adult primary care patients who completed screening and/or assessment measures also allowed us to obtain a population-representative sample that likely had limited sampling bias (i.e., the sample was not restricted to people who would choose to participate in an alcohol- and depression-related research study). Screening and assessment rates were high, and the large sample sizes available for analysis allowed us to obtain a strong degree of precision in our analyses. 

## Conclusion

In this population-based sample of primary care patients who completed screenings for alcohol use and depression as part of routine care, probable DSM-5 AUD was common in patients with high risk drinking, particularly when they also screened positive for depression. For adult patients who screen positive for high-risk drinking and depression, assessing AUD symptoms using an Alcohol Symptom Checklist may help support alcohol and depression care management by identifying the high proportion of patients who could likely benefit from AUD treatments that can be offered in primary care.

## Supplementary Information

**Additional file 1: eTable 1.** Comparison of primary care patients with versus without an AUDIT-C screen completed during the study period. **eTable 2.** Comparison of high-risk drinking primary care patients with versus without an Alcohol Symptom Checklist completed during the study period. **eTable 3.** Associations of scaled PHQ-2 depression screening scores (0-6) with high-risk drinking on the AUDIT-C. **eTable 4.** Associations of binary PHQ-2 depression screening scores (cutoff score: 3) with high-risk drinking on the AUDIT-C. **eTable 5.** Associations of binary PHQ-2 depression screening scores (cutoff score: 2) with high-risk drinking on the AUDIT-C. **eTable 6.** Associations of scaled PHQ-2 depression screening scores (0-6) with probable AUD (Alcohol Symptom Checklist scores 2-11). **eTable 7.** Associations of binary PHQ-2 depression screening scores (cutoff score: 3) with probable AUD (Alcohol Symptom Checklist scores 2-11). **eTable 8.** Associations of binary PHQ-2 depression screening scores (cutoff score: 2) with probable AUD (Alcohol Symptom Checklists scores 0-11). **eFigure 1.** Prevalence of high-risk drinking across PHQ-2 depression screening scores, stratified by patient sex. **eFigure 2.** Prevalence of probable AUD (mild, moderate, or severe) across PHQ-2 depression screening scores, stratified by patient sex in the checklist subsample (patients with high-risk drinking).

## Data Availability

The datasets generated and analyzed for the current study are not publicly available due to the potential for datasets to compromise patient privacy and due to data use agreements that prohibit sharing clinical data that was originally generated for clinical (i.e., non-research) purposes with third parties. Aggregate data supporting the findings of this study are available from the corresponding author upon request.

## Abbreviations

AUD: Alcohol Use Disorder
AUDIT-C: Alcohol Use Disorders Identification Test—Consumption Version
DSM-5: Diagnostic and Statistical Manual for Mental Disorders, 5^th^ edition
EHR: Electronic health record
GLM: Generalized linear model
KPWA: Kaiser Permanente Washington
PHQ: Patient Health Questionnaire
