# Supplementary material for: An Alcohol Symptom Checklist identifies high rates of alcohol use disorder in primary care patients who screen positive for depression and high-risk drinking
Source: BMC Health Serv Res. 2022 Sep 5;22:1123. doi: 10.1186/s12913-022-08408-1 (PMC9446862; doi:10.1186/s12913-022-08408-1)

Supplement to:

**An Alcohol Symptom Checklist Identifies High Rates of Alcohol Use Disorder in Primary Care Patients Who Screen Positive for Depression and High-Risk Drinking**

**Contents:**

[eTable 1. Comparison of primary care patients with versus without an AUDIT-C screen completed during the study period. 2](#_Toc105491905)

[eTable 2. Comparison of high-risk drinking primary care patients with versus without an Alcohol Symptom Checklist completed during the study period. 3](#_Toc105491906)

[eTable 3. Associations of scaled PHQ-2 depression screening scores (0-6) with high-risk drinking on the AUDIT-C 4](#_Toc105491907)

[eTable 4. Associations of binary PHQ-2 depression screening scores (cutoff score: 3) with high-risk drinking on the AUDIT-C 5](#_Toc105491908)

[eTable 5. Associations of binary PHQ-2 depression screening scores (cutoff score: 2) with high-risk drinking on the AUDIT-C 6](#_Toc105491909)

[eTable 6. Associations of scaled PHQ-2 depression screening scores (0-6) with probable AUD (Alcohol Symptom Checklist scores 2-11) 7](#_Toc105491910)

[eTable 7. Associations of binary PHQ-2 depression screening scores (cutoff score: 3) with probable AUD (Alcohol Symptom Checklist scores 2-11) 8](#_Toc105491911)

[eTable 8. Associations of binary PHQ-2 depression screening scores (cutoff score: 2) with probable AUD (Alcohol Symptom Checklists scores 0-11) 9](#_Toc105491912)

[eFigure 1. Prevalence of high-risk drinking across PHQ-2 depression screening scores, stratified by patient sex. 10](#_Toc105491913)

[eFigure 2. Prevalence of probable AUD (mild, moderate, or severe) across PHQ-2 depression screening scores, stratified by patient sex in the checklist subsample (patients with high-risk drinking). 11](#_Toc105491914)

# eTable 1. Comparison of primary care patients with versus without an AUDIT-C screen completed during the study period.

|  | Completed AUDIT-C (N=369,937) | | Did not complete AUDIT-C (N=50,818) | |  |
| --- | --- | --- | --- | --- | --- |
|  | n | % | n | % | p-value |
| **Sex** |  |  |  |  |  |
| Female | 216958 | 58.6% | 28024 | 55.1% | <.001 |
| Male | 152977 | 41.4% | 22794 | 44.9% |  |
| Unknown | 2 | 0.0% | 0 | 0.0% |  |
| **Race** |  |  |  |  |  |
| Asian or Asian American | 35564 | 9.6% | 4653 | 9.2% | <.001 |
| Black or African American | 17362 | 4.7% | 2122 | 4.2% |  |
| Native Hawaiian/Pacific Islander | 3519 | 1.0% | 489 | 1.0% |  |
| American Indian/Alaska Native | 2580 | 0.7% | 410 | 0.8% |  |
| White | 266440 | 72.0% | 35589 | 70.0% |  |
| More than one race | 10352 | 2.8% | 1387 | 2.7% |  |
| Other race | 14768 | 4.0% | 2056 | 4.0% |  |
| Unknown | 19352 | 5.2% | 4112 | 8.1% |  |
| **Ethnicity** |  |  |  |  |  |
| Non-Hispanic | 327997 | 88.7% | 43655 | 85.9% | <.001 |
| Unknown | 19822 | 5.4% | 4298 | 8.5% |  |
| Hispanic | 22118 | 6.0% | 2865 | 5.6% |  |
| **Age** |  |  |  |  |  |
| 18-24 | 32349 | 8.7% | 6072 | 11.9% | <.001 |
| 25-44 | 118298 | 32.0% | 17040 | 33.5% |  |
| 45-64 | 134467 | 36.3% | 18585 | 36.6% |  |
| 65+ | 84823 | 22.9% | 9121 | 17.9% |  |

Note: Sample sizes reported in the main text reflect the demographic information that was available in the electronic health record at the time AUDIT-C screens were completed. Sample sizes reported in this table reflect the demographic information that was available in the electronic health record on the last day of the study period. Therefore, subsample n’s may differ slightly in this table compared to what is presented in the main text.

# eTable 2. Comparison of high-risk drinking primary care patients with versus without an Alcohol Symptom Checklist completed during the study period.

|  | Completed Alcohol Symptom Checklist (N=8184) | | Did not complete Alcohol Symptom Checklist (N=2851) | |  |
| --- | --- | --- | --- | --- | --- |
|  | n | % | n | % | p-value |
| **Sex** |  |  |  |  |  |
| Female | 2531 | 30.9% | 818 | 28.7% | 0.03 |
| Male | 5653 | 69.1% | 2033 | 71.3% |  |
| **Race** |  |  |  |  |  |
| Asian or Asian American | 386 | 4.7% | 166 | 5.8% | 0.01 |
| Black or African American | 401 | 4.9% | 121 | 4.2% |  |
| Native Hawaiian/Pacific Islander | 90 | 1.1% | 34 | 1.2% |  |
| American Indian/Alaska Native | 82 | 1.0% | 24 | 0.8% |  |
| **White** | 6161 | 75.3% | 2091 | 73.3% |  |
| More than one race | 221 | 2.7% | 93 | 3.3% |  |
| Other race | 394 | 4.8% | 128 | 4.5% |  |
| Unknown | 449 | 5.5% | 194 | 6.8% |  |
| Ethnicity |  |  |  |  |  |
| Non-Hispanic | 7133 | 87.2% | 2434 | 85.4% | 0.001 |
| Unknown | 474 | 5.8% | 221 | 7.8% |  |
| Hispanic | 577 | 7.1% | 196 | 6.9% |  |
| **Age** |  |  |  |  |  |
| 18-24 | 903 | 11.0% | 311 | 10.9% | 0.06 |
| 25-44 | 3532 | 43.2% | 1272 | 44.6% |  |
| 45-64 | 2927 | 35.8% | 1030 | 36.1% |  |
| 65+ | 822 | 10.0% | 238 | 8.3% |  |
| AUDIT-C score, mean (SD) | 7.46 | (2.14) | 7.94 | (1.24) | <0.001 |

Note: Sample sizes reported in the main text reflect the demographic information that was available in the electronic health record at the time AUDIT-C screens were completed. Sample sizes reported in this table reflect the demographic information that was available in the electronic health record on the last day of the study period. Therefore, subsample n’s may differ slightly in this table compared to what is presented in the main text.

# eTable 3. Associations of scaled PHQ-2 depression screening scores (0-6) with high-risk drinking on the AUDIT-C

|  | Coefficient | SE | t-value | p-value | aPR | (aPR 95% CI) | |
| --- | --- | --- | --- | --- | --- | --- | --- |
| (Intercept) | -3.78 | 0.07 | -54.75 | <.001 | 0.02 | (0.02, | 0.03) |
| **Positive PHQ-2 Score (scaled)** | **0.24** | **0.01** | **47.37** | **<.001** | **1.27** | **(1.26,** | **1.29)** |
| Sex (female) | -1.34 | 0.02 | -60.45 | <.001 | 0.26 | (0.25, | 0.27) |
| Race (Black/African American) | 0.50 | 0.06 | 7.84 | <.001 | 1.65 | (1.46, | 1.88) |
| Race (Native Hawaiian/Pacific Islander) | 0.67 | 0.10 | 6.58 | <.001 | 1.96 | (1.61, | 2.40) |
| Race (American Indian/Alaska Native) | 0.87 | 0.11 | 7.86 | <.001 | 2.39 | (1.92, | 2.97) |
| Race (White) | 0.69 | 0.05 | 14.86 | <.001 | 1.99 | (1.82, | 2.18) |
| Race (Multiracial) | 0.47 | 0.08 | 6.26 | <.001 | 1.61 | (1.39, | 1.86) |
| Race (Other) | 0.60 | 0.07 | 8.88 | <.001 | 1.81 | (1.59, | 2.07) |
| Race (Unknown) | 0.41 | 0.09 | 4.74 | <.001 | 1.50 | (1.27, | 1.77) |
| Ethnicity (not Hispanic) | -0.08 | 0.04 | -1.82 | 0.07 | 0.93 | (0.85, | 1.01) |
| Ethnicity (Unknown) | 0.05 | 0.08 | 0.61 | 0.54 | 1.05 | (0.90, | 1.23) |
| Age (25-44) | 0.20 | 0.03 | 5.77 | <.001 | 1.22 | (1.14, | 1.31) |
| Age (45-64) | -0.11 | 0.04 | -3.02 | <.001 | 0.90 | (0.84, | 0.96) |
| Age (65+) | -1.03 | 0.05 | -22.59 | <.001 | 0.36 | (0.33, | 0.39) |

Note: Dependent variable is a binary outcome reflecting high-risk drinking, defined by AUDIT-C scores of 7-12 (1) versus AUDIT-C scores of 0-6 (0). For each demographic variable, one subgroup was selected as the reference group for whom no coefficients are estimated: age (18-24), sex (male), race (Asian American), and ethnicity (Hispanic). aPR = adjusted prevalence ratio.

# eTable 4. Associations of binary PHQ-2 depression screening scores (cutoff score: 3) with high-risk drinking on the AUDIT-C

|  | Coefficient | SE | t-value | p-value | aPR | (aPR 95% CI) | |
| --- | --- | --- | --- | --- | --- | --- | --- |
| (Intercept) | -3.63 | 0.07 | -52.71 | <.001 | 0.03 | (0.02, | 0.03) |
| **Positive PHQ-2 Score (≥3)** | **0.84** | **0.02** | **36.95** | **<.001** | **2.31** | **(2.21,** | **2.42)** |
| Sex (female) | -1.32 | 0.02 | -59.39 | <.001 | 0.27 | (0.26, | 0.28) |
| Race (Black/African American) | 0.52 | 0.06 | 8.05 | <.001 | 1.68 | (1.48, | 1.90) |
| Race (Native Hawaiian/Pacific Islander) | 0.69 | 0.10 | 6.72 | <.001 | 1.99 | (1.63, | 2.43) |
| Race (American Indian/Alaska Native) | 0.91 | 0.11 | 8.25 | <.001 | 2.49 | (2.01, | 3.10) |
| Race (White) | 0.71 | 0.05 | 15.37 | <.001 | 2.04 | (1.86, | 2.23) |
| Race (Multiracial) | 0.50 | 0.08 | 6.64 | <.001 | 1.66 | (1.43, | 1.92) |
| Race (Other) | 0.61 | 0.07 | 9.11 | <.001 | 1.85 | (1.62, | 2.11) |
| Race (Unknown) | 0.42 | 0.09 | 4.91 | <.001 | 1.52 | (1.29, | 1.80) |
| Ethnicity (not Hispanic) | -0.08 | 0.04 | -1.90 | 0.06 | 0.92 | (0.85, | 1.00) |
| Ethnicity (Unknown) | 0.05 | 0.08 | 0.58 | 0.56 | 1.05 | (0.90, | 1.22) |
| Age (25-44) | 0.18 | 0.03 | 5.29 | <.001 | 1.20 | (1.12, | 1.28) |
| Age (45-64) | -0.15 | 0.04 | -4.34 | <.001 | 0.86 | (0.80, | 0.92) |
| Age (65+) | -1.09 | 0.05 | -23.97 | <.001 | 0.34 | (0.31, | 0.37) |

Note: Dependent variable is a binary outcome reflecting high-risk drinking, defined by AUDIT-C scores of 7-12 (1) versus AUDIT-C scores of 0-6 (0). For each demographic variable, one subgroup was selected as the reference group for whom no coefficients are estimated: age (18-24), sex (male), race (Asian American), and ethnicity (Hispanic). aPR = adjusted prevalence ratio.

# eTable 5. Associations of binary PHQ-2 depression screening scores (cutoff score: 2) with high-risk drinking on the AUDIT-C

|  | Coefficient | SE | t-value | p-value | aPR | (aPR 95% CI) | |
| --- | --- | --- | --- | --- | --- | --- | --- |
| (Intercept) | -3.75 | 0.07 | -54.13 | <.001 | 0.02 | (0.02, | 0.03) |
| **Positive PHQ-2 Score (≥2)** | **0.78** | **0.02** | **39.04** | **<.001** | **2.18** | **(2.10,** | **2.27)** |
| Sex (female) | -1.33 | 0.02 | -60.08 | <.001 | 0.26 | (0.25, | 0.28) |
| Race (Black/African American) | 0.53 | 0.06 | 8.20 | <.001 | 1.69 | (1.49, | 1.92) |
| Race (Native Hawaiian/Pacific Islander) | 0.69 | 0.10 | 6.68 | <.001 | 1.99 | (1.62, | 2.43) |
| Race (American Indian/Alaska Native) | 0.91 | 0.11 | 8.22 | <.001 | 2.48 | (2.00, | 3.08) |
| Race (White) | 0.70 | 0.05 | 15.15 | <.001 | 2.02 | (1.84, | 2.21) |
| Race (Multiracial) | 0.50 | 0.08 | 6.55 | <.001 | 1.64 | (1.42, | 1.90) |
| Race (Other) | 0.61 | 0.07 | 9.08 | <.001 | 1.84 | (1.61, | 2.10) |
| Race (Unknown) | 0.43 | 0.09 | 5.06 | <.001 | 1.54 | (1.30, | 1.82) |
| Ethnicity (not Hispanic) | -0.08 | 0.04 | -1.78 | 0.07 | 0.93 | (0.85, | 1.01) |
| Ethnicity (Unknown) | 0.04 | 0.08 | 0.46 | 0.65 | 1.04 | (0.89, | 1.21) |
| Age (25-44) | 0.18 | 0.03 | 5.15 | <.001 | 1.19 | (1.12, | 1.28) |
| Age (45-64) | -0.13 | 0.04 | -3.69 | <.001 | 0.88 | (0.82, | 0.94) |
| Age (65+) | -1.05 | 0.05 | -23.21 | <.001 | 0.35 | (0.32, | 0.38) |

Note: Dependent variable is a binary outcome reflecting high-risk drinking, defined by AUDIT-C scores of 7-12 (1) versus AUDIT-C scores of 0-6 (0). For each demographic variable, one subgroup was selected as the reference group for whom no coefficients are estimated: age (18-24), sex (male), race (Asian American), and ethnicity (Hispanic). aPR = adjusted prevalence ratio.

# eTable 6. Associations of scaled PHQ-2 depression screening scores (0-6) with probable AUD (Alcohol Symptom Checklist scores 2-11)

|  | Coefficient | SE | t-value | p-value | aPR | (aPR 95% CI) | |
| --- | --- | --- | --- | --- | --- | --- | --- |
| (Intercept) | -1.06 | 0.08 | -13.75 | <.001 | 0.35 | (0.30, | 0.40) |
| **Positive PHQ-2 Score (scaled)** | **0.12** | **0.00** | **24.86** | **<.001** | **1.12** | **(1.11,** | **1.13)** |
| Sex (female) | 0.04 | 0.02 | 2.13 | 0.03 | 1.05 | (1.00, | 1.09) |
| Race (Black/African American) | 0.14 | 0.07 | 1.95 | 0.05 | 1.15 | (1.00, | 1.33) |
| Race (Native Hawaiian/Pacific Islander) | -0.18 | 0.14 | -1.29 | 0.20 | 0.83 | (0.63, | 1.10) |
| Race (American Indian/Alaska Native) | 0.19 | 0.11 | 1.62 | 0.11 | 1.20 | (0.96, | 1.51) |
| Race (White) | 0.22 | 0.06 | 3.74 | <.001 | 1.24 | (1.11, | 1.39) |
| Race (Multiracial) | 0.16 | 0.08 | 2.02 | 0.04 | 1.18 | (1.00, | 1.38) |
| Race (Other) | 0.16 | 0.08 | 2.06 | 0.04 | 1.17 | (1.01, | 1.37) |
| Race (Unknown) | 0.19 | 0.10 | 1.91 | 0.06 | 1.21 | (1.00, | 1.46) |
| Ethnicity (not Hispanic) | 0.00 | 0.04 | 0.04 | 0.97 | 1.00 | (0.92, | 1.09) |
| Ethnicity (Unknown) | -0.04 | 0.09 | -0.46 | 0.65 | 0.96 | (0.81, | 1.14) |
| Age (25-44) | 0.07 | 0.03 | 2.22 | 0.03 | 1.07 | (1.01, | 1.15) |
| Age (45-64) | -0.02 | 0.03 | -0.60 | 0.55 | 0.98 | (0.91, | 1.05) |
| Age (65+) | -0.12 | 0.05 | -2.46 | 0.01 | 0.89 | (0.81, | 0.98) |

Note: Dependent variable is a binary outcome reflecting probable AUD, defined by Alcohol Symptom Checklist scores of 2-11 (1) versus Alcohol Symptom Checklist scores of 0-1 (0). For each demographic variable, one subgroup was selected as the reference group for whom no coefficients are estimated: age (18-24), sex (male), race (Asian American), and ethnicity (Hispanic). aPR = adjusted prevalence ratio.

# eTable 7. Associations of binary PHQ-2 depression screening scores (cutoff score: 3) with probable AUD (Alcohol Symptom Checklist scores 2-11)

|  | Coefficient | SE | t-value | p-value | aPR | (aPR 95% CI) | |
| --- | --- | --- | --- | --- | --- | --- | --- |
| (Intercept) | -0.95 | 0.08 | -12.26 | <.001 | 0.39 | (0.33, | 0.45) |
| **Positive PHQ-2 Score (≥3)** | **0.35** | **0.02** | **17.59** | **<.001** | **1.42** | **(1.36,** | **1.47)** |
| Sex (female) | 0.08 | 0.02 | 4.05 | <.001 | 1.09 | (1.04, | 1.13) |
| Race (Black/African American) | 0.15 | 0.07 | 2.09 | 0.04 | 1.17 | (1.01, | 1.35) |
| Race (Native Hawaiian/Pacific Islander) | -0.19 | 0.14 | -1.34 | 0.18 | 0.83 | (0.63, | 1.09) |
| Race (American Indian/Alaska Native) | 0.22 | 0.12 | 1.91 | 0.06 | 1.25 | (0.99, | 1.56) |
| Race (White) | 0.23 | 0.06 | 3.92 | <.001 | 1.26 | (1.12, | 1.41) |
| Race (Multiracial) | 0.19 | 0.08 | 2.22 | 0.03 | 1.20 | (1.02, | 1.42) |
| Race (Other) | 0.17 | 0.08 | 2.20 | 0.03 | 1.19 | (1.02, | 1.39) |
| Race (Unknown) | 0.20 | 0.10 | 2.04 | 0.04 | 1.22 | (1.01, | 1.48) |
| Ethnicity (not Hispanic) | 0.00 | 0.05 | -0.10 | 0.92 | 1.00 | (0.91, | 1.09) |
| Ethnicity (Unknown) | -0.03 | 0.09 | -0.39 | 0.69 | 0.97 | (0.81, | 1.15) |
| Age (25-44) | 0.07 | 0.03 | 2.04 | 0.04 | 1.07 | (1.00, | 1.14) |
| Age (45-64) | -0.05 | 0.04 | -1.33 | 0.18 | 0.95 | (0.89, | 1.02) |
| Age (65+) | -0.16 | 0.05 | -3.27 | <.001 | 0.85 | (0.78, | 0.94) |

Note: Dependent variable is a binary outcome reflecting probable AUD, defined by Alcohol Symptom Checklist scores of 2-11 (1) versus Alcohol Symptom Checklist scores of 0-1 (0). For each demographic variable, one subgroup was selected as the reference group for whom no coefficients are estimated: age (18-24), sex (male), race (Asian American), and ethnicity (Hispanic). aPR = adjusted prevalence ratio.

# eTable 8. Associations of binary PHQ-2 depression screening scores (cutoff score: 2) with probable AUD (Alcohol Symptom Checklists scores 0-11)

|  | Coefficient | SE | t-value | p-value | aPR | (aPR 95% CI) | |
| --- | --- | --- | --- | --- | --- | --- | --- |
| (Intercept) | -1.07 | 0.08 | -13.75 | <.001 | 0.34 | (0.30, | 0.40) |
| **Positive PHQ-2 Score (≥2)** | **0.47** | **0.02** | **21.64** | **<.001** | **1.60** | **(1.53,** | **1.67)** |
| Sex (female) | 0.05 | 0.02 | 2.59 | 0.01 | 1.05 | (1.01, | 1.10) |
| Race (Black/African American) | 0.15 | 0.07 | 2.10 | 0.04 | 1.16 | (1.01, | 1.34) |
| Race (Native Hawaiian/Pacific Islander) | -0.14 | 0.14 | -1.01 | 0.31 | 0.87 | (0.66, | 1.14) |
| Race (American Indian/Alaska Native) | 0.17 | 0.12 | 1.45 | 0.15 | 1.19 | (0.94, | 1.49) |
| Race (White) | 0.22 | 0.06 | 3.78 | <.001 | 1.25 | (1.11, | 1.39) |
| Race (Multiracial) | 0.15 | 0.08 | 1.79 | 0.07 | 1.16 | (0.99, | 1.36) |
| Race (Other) | 0.14 | 0.08 | 1.79 | 0.07 | 1.15 | (0.99, | 1.34) |
| Race (Unknown) | 0.19 | 0.10 | 1.96 | 0.05 | 1.21 | (1.00, | 1.47) |
| Ethnicity (not Hispanic) | -0.01 | 0.04 | -0.27 | 0.79 | 0.99 | (0.91, | 1.08) |
| Ethnicity (Unknown) | -0.06 | 0.09 | -0.68 | 0.50 | 0.94 | (0.79, | 1.12) |
| Age (25-44) | 0.06 | 0.03 | 1.71 | 0.09 | 1.06 | (0.99, | 1.13) |
| Age (45-64) | -0.03 | 0.03 | -0.97 | 0.33 | 0.97 | (0.90, | 1.03) |
| Age (65+) | -0.13 | 0.05 | -2.80 | 0.01 | 0.88 | (0.80, | 0.96) |

Note: Dependent variable is a binary outcome reflecting probable AUD, defined by Alcohol Symptom Checklist scores of 2-11 (1) versus Alcohol Symptom Checklist scores of 0-1 (0). For each demographic variable, one subgroup was selected as the reference group for whom no coefficients are estimated: age (18-24), sex (male), race (Asian American), and ethnicity (Hispanic). aPR = adjusted prevalence ratio.

# eFigure 1. Prevalence of high-risk drinking across PHQ-2 depression screening scores, stratified by patient sex.


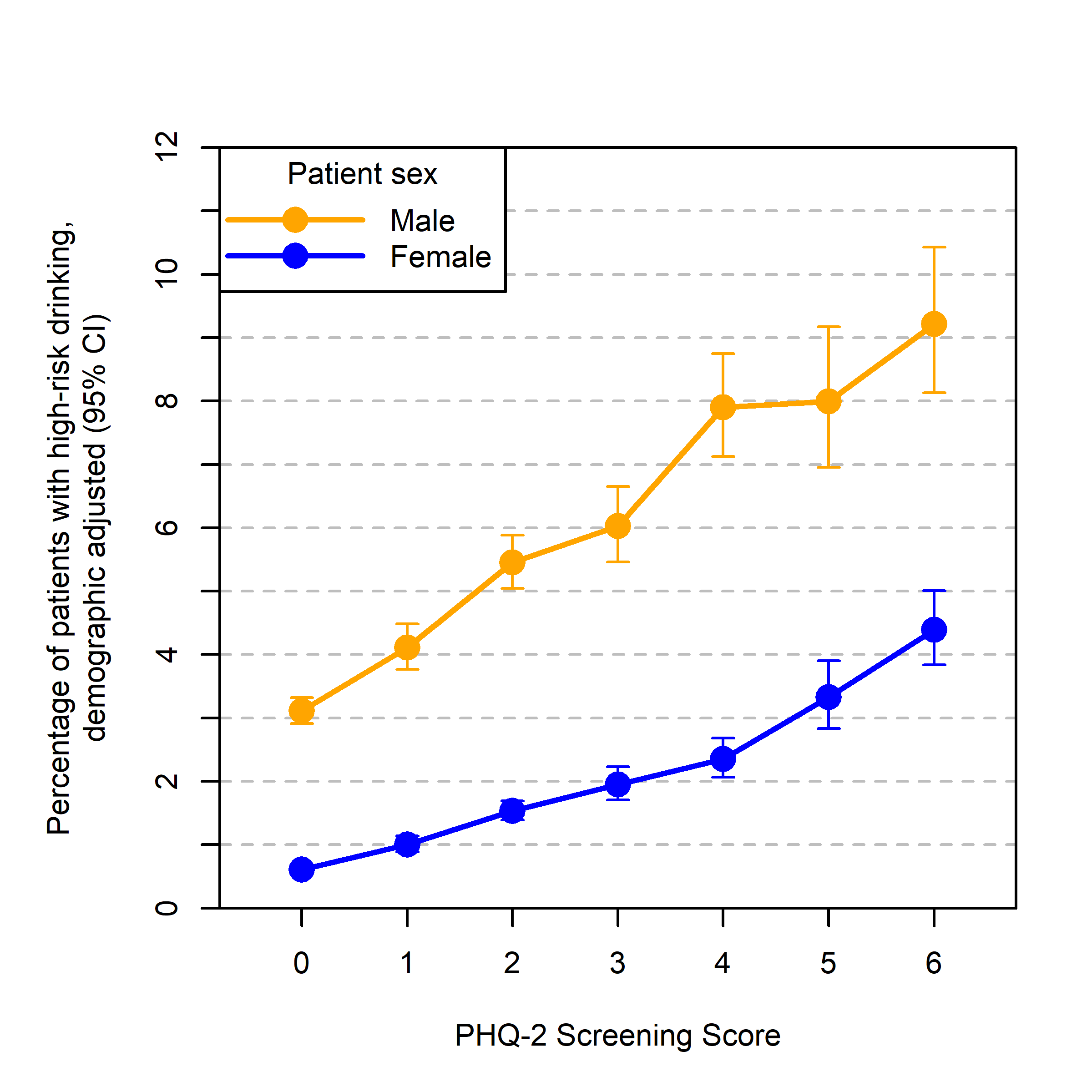


# eFigure 2. Prevalence of probable AUD (mild, moderate, or severe) across PHQ-2 depression screening scores, stratified by patient sex in the checklist subsample (patients with high-risk drinking).


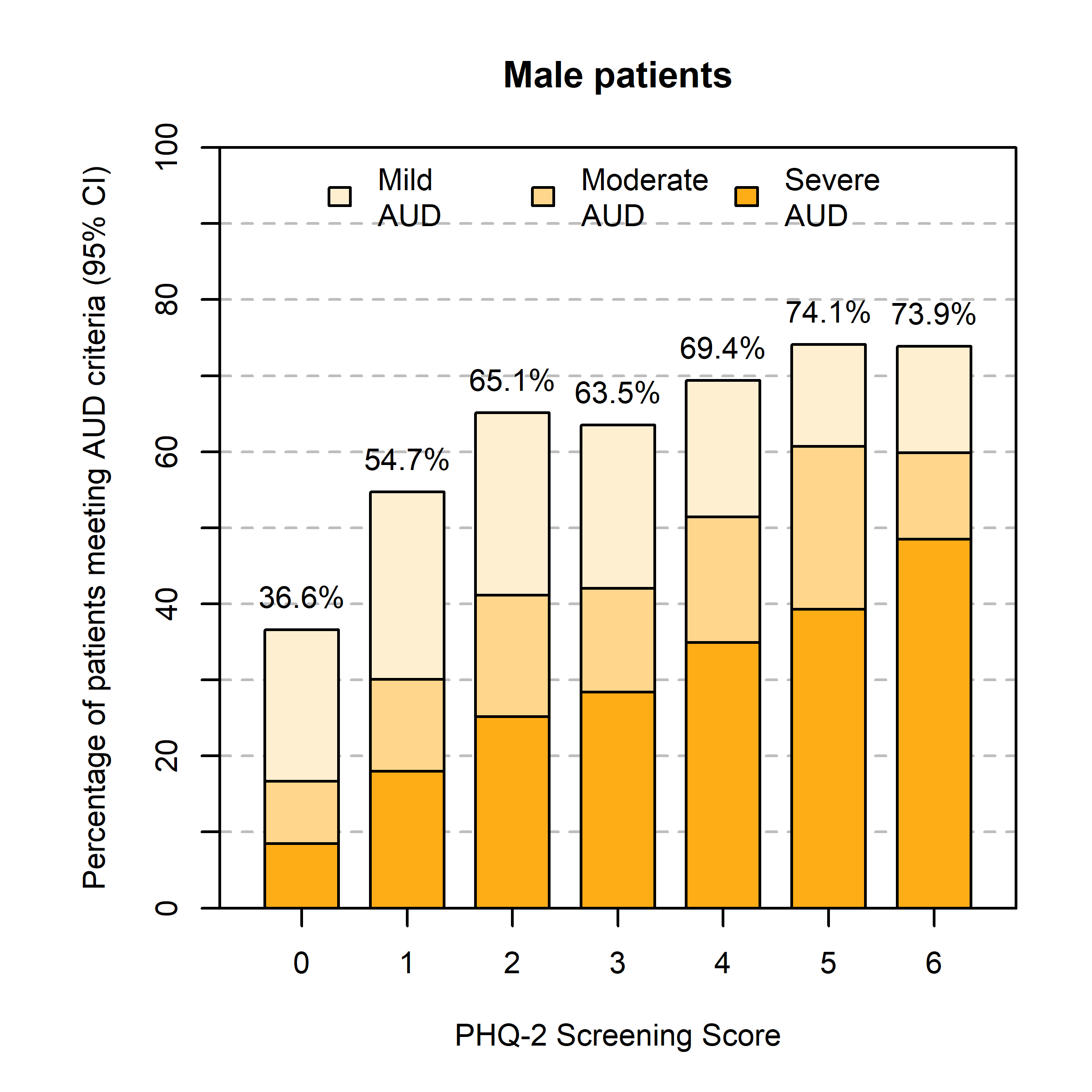

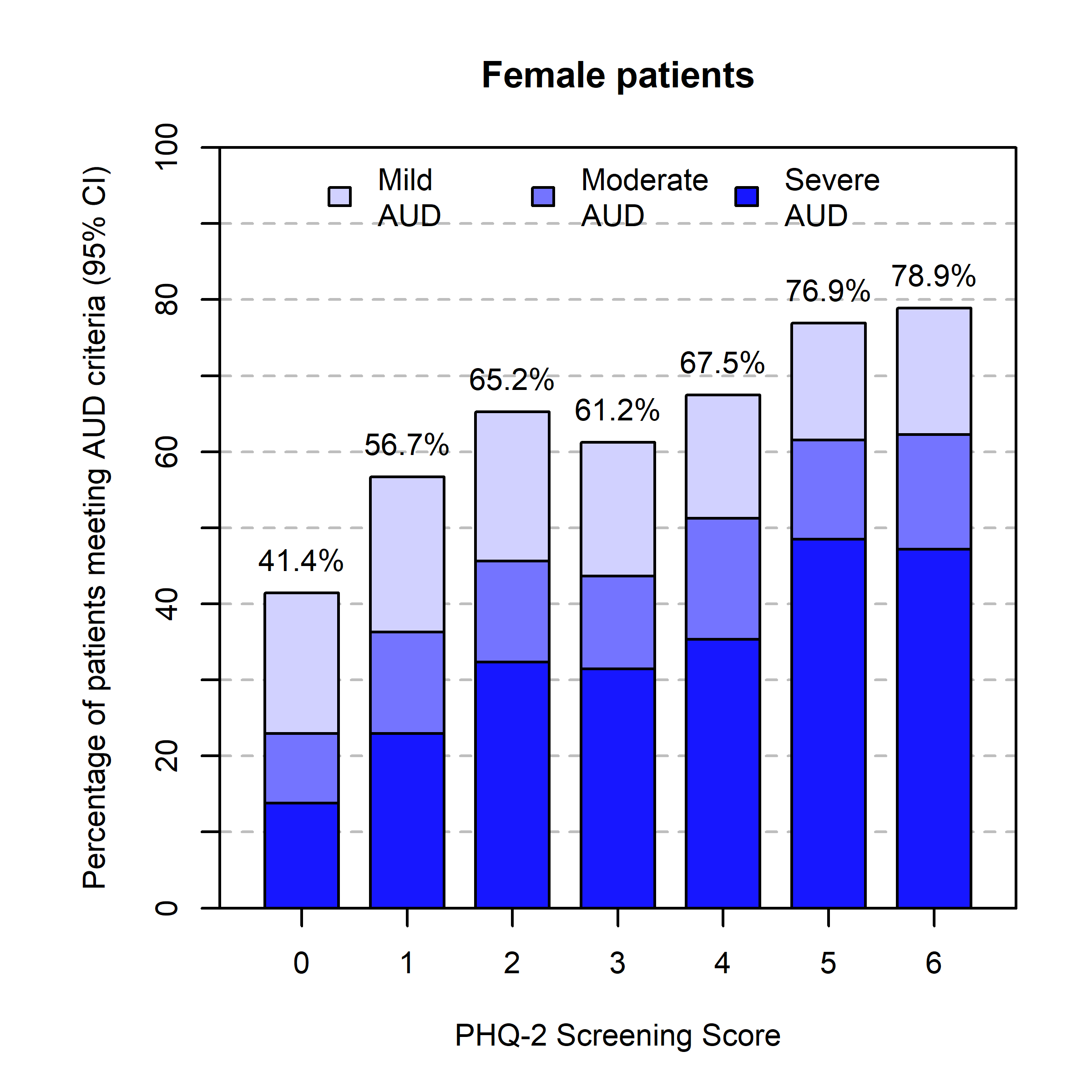

Supplement: Supplementary file 1 — Additional file 1: eTable 1. Comparison of primary care patients with versus without an AUDIT-C screen completed during the study period. eTable 2. Comparison of high-risk drinking primary care patients with versus without an Alcohol Symptom Checklist completed during the study period. eTable 3. Associations of scaled PHQ-2 depression screening scores (0-6) with high-risk drinking on the AUDIT-C. eTable 4. Associations of binary PHQ-2 depression screening scores (cutoff score: 3) with high-risk drinking on the AUDIT-C. eTable 5. Associations of binary PHQ-2 depression screening scores (cutoff score: 2) with high-risk drinking on the AUDIT-C. eTable 6. Associations of scaled PHQ-2 depression screening scores (0-6) with probable AUD (Alcohol Symptom Checklist scores 2-11). eTable 7. Associations of binary PHQ-2 depression screening scores (cutoff score: 3) with probable AUD (Alcohol Symptom Checklist scores 2-11). eTable 8. Associations of binary PHQ-2 depression screening scores (cutoff score: 2) with probable AUD (Alcohol Symptom Checklists scores 0-11). eFigure 1. Prevalence of high-risk drinking across PHQ-2 depression screening scores, stratified by patient sex. eFigure 2. Prevalence of probable AUD (mild, moderate, or severe) across PHQ-2 depression screening scores, stratified by patient sex in the checklist subsample (patients with high-risk drinking). [file 12913_2022_8408_MOESM1_ESM.docx]
